# Author Correction: Bismuthene for highly efficient carbon dioxide electroreduction reaction

**DOI:** 10.1038/s41467-020-16260-2

**Published:** 2020-05-06

**Authors:** Fa Yang, Ahmed O. Elnabawy, Roberto Schimmenti, Ping Song, Jiawei Wang, Zhangquan Peng, Shuang Yao, Ruiping Deng, Shuyan Song, Yue Lin, Manos Mavrikakis, Weilin Xu

**Affiliations:** 1 State Key Laboratory of Electroanalytical Chemistry, & Jilin Province Key Laboratory of Low Carbon Chemical Power, Changchun Institute of Applied Chemistry, Chinese Academy of Sciences, 5625 Renmin Street, 130022 Changchun, P.R. China; 2 University of Science and Technology of China, 230026 Anhui, P.R. China; 3 Department of Chemical & Biological Engineering, University of Wisconsin-Madison, Madison, WI 53706 USA; 4 State Key Laboratory of Rare Earth, Changchun Institute of Applied Chemistry, Chinese Academy of Sciences, 5625 Renmin Street, 130022 Changchun, P.R. China

**Keywords:** Heterogeneous catalysis, Electrocatalysis, Two-dimensional materials

Correction to: *Nature Communications* 10.1038/s41467-020-14914-9, published online 27 February 2020

The original version of this Article contained an error in the author affiliations.

The affiliation of Weilin Xu with ‘University of Science and Technology of China, 230026 Anhui, P.R. China’ was inadvertently omitted.

This has now been corrected in both the PDF and HTML versions of the Article.

