# Supplementary material for: Bismuthene for highly efficient carbon dioxide electroreduction reaction
Source: Nat Commun. 2020 Feb 27;11:1088. doi: 10.1038/s41467-020-14914-9 (PMC7046785; doi:10.1038/s41467-020-14914-9)
Supplement: Supplementary file 2 — Supplementary Information [file 41467_2020_14914_MOESM2_ESM.pdf]

SUPPLEMENTARY INFORMATION

Bismuthene for highly efficient carbon dioxide electroreduction reaction

Fa Yang *et al*.

Supplementary Note 1

In order to describe the zig-zag arrangement for Bi atoms in the Bi(111) facet as shown in Fig. 1b,f, the atoms in the first layer were colored in blue, and those in the second layer in purple (Supplementary Fig. 3a,b). When the structure was rotated 45 º (Supplementary Fig. 3c) and 90 º down (Supplementary Fig. 3d), the hexagonal pattern formed by the Bi atoms in the first and second layer is evident. Actually, they form a typical hexagonal chair structure in 3D space (Supplementary Fig. 3f). In the HRTEM image (Supplementary Fig. 3e), one can find a distribution of bright spots, corresponding to Bi atoms in the first layer, around dark spots, corresponding to Bi atoms in the second layer. This specific pattern forms the zig-zag structures in Supplementary Fig. 3a and Fig. 1f. Based on such features, one can also find a hexagon-like projection (the green one in the inset of Supplementary Fig. 3e), and the real three-dimensional structure is chair-like hexagonal, as shown in Supplementary Fig. 3f. So, for the Bismuthene monolayer obtained here, the real space configuration is indeed a honeycomb hexagonal arrangement, while in HRTEM, due to the fact that the upper atoms are projected exactly on either side of the lower atoms, a quadrilateral-like arrangement of Bi atoms could be observed apparently in 2D HRTEM image.

Supplementary Note 2

CO_2 Temperature-programmed desorption (CO_2-TPD) was further carried out to investigate the CO_2 adsorption capability of these samples. As shown in Supplementary Fig. 11, the desorption peak centered at 121 ^oC could be assigned to the physisorption, and the wide desorption centered at 310 ^oC (0.65 nm), 375 ^oC (4.2 nm), and 395 ^oC (11.3 nm) corresponded to the chemisorption of CO_2. It is worth noting that 0.65 nm-nanosheets exhibit the largest CO_2 desorption peak area, followed by 4.2 nm and 11.3 nm, implying that the monolayer Bismuthene nanosheets indeed have larger CO_2 adsorption capability compared with the thicker nanosheets, which is consistent with CO_2 adsorption isotherms of BiNSs with different thickness shown in Supplementary Fig. 10.

Supplementary Note 3

As for the stability of matrix structure of the Bismuthene, we did the HRTEM analysis of the monolayer Bismuthene after long-term (75 hrs) high current density operation (at fixed potential of -0.88 V vs. RHE). As shown in above Supplementary Fig. 21, after long-term high current density operation, the lattice matrix structure shows almost no change. Such fact further confirms the remarkable durability of monolayer Bismuthene during the CO2RR process, just like that shown by TEM (Supplementary Fig. 19) and Raman (Supplementary Figs. 17-18) analysis.

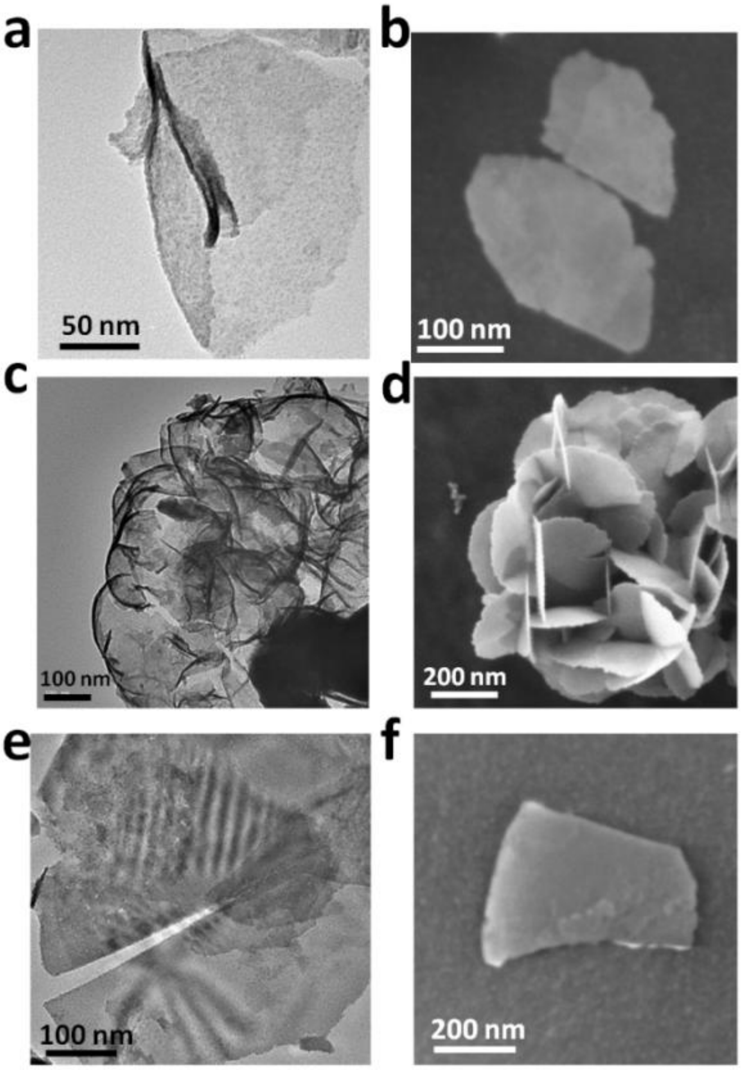

Supplementary Fig. 1 Typical TEM (a,c,e) and SEM (b,d,f) images of BiNSs with different average thickness <H>: a,b) <H>=0.65 nm, c,d) <H>=4.2 nm and e,f) <H>=11.3 nm.

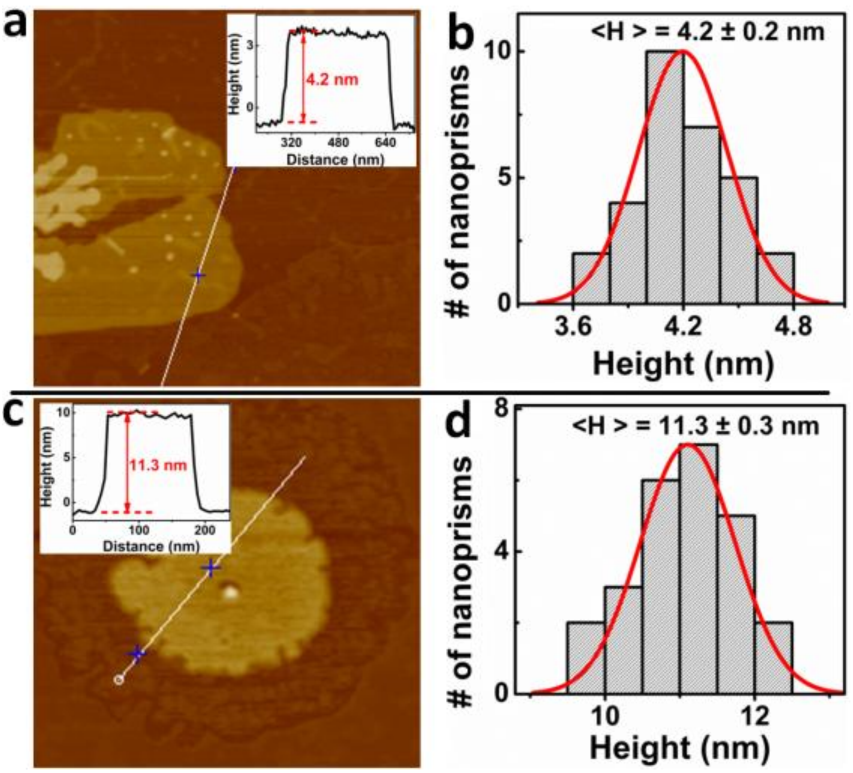

Supplementary Fig. 2 Typical atomic force microscopy (AFM) image and the corresponding height profiles for BiNSs with average thickness of a, b) 4.2 nm; c, d) 11.3 nm.

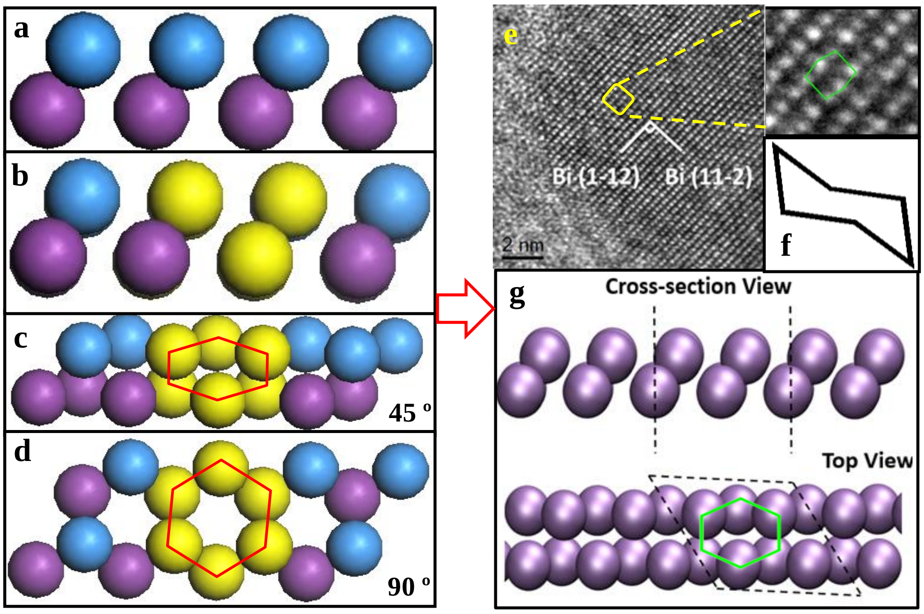

Supplementary Fig. 3 The structure arrangement for Bi(111) ((a) and (b)), and rotated 45 º(c) and 90 º down (d); (e) HRTEM image; the honeycomb hexagonal arrangement in yellow area is reported in the inset; (f) Space configuration for the honeycomb hexagonal arrangement in Figure S3e; (g) Honeycomb hexagonal arrangement (green ring) in the optimized geometry, at the PBE+TS level, of a Bismuthene monolayer.

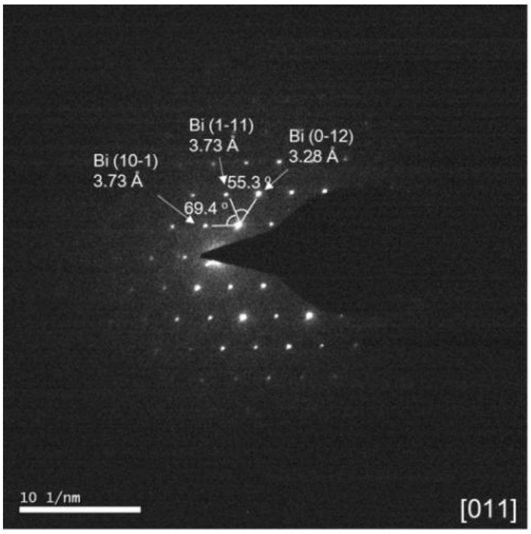

Supplementary Fig. 4 Typical SAED image of thick Bi nanosheet with thickness about 4.2 nm indicates the exposure of [011] facet on its surface.

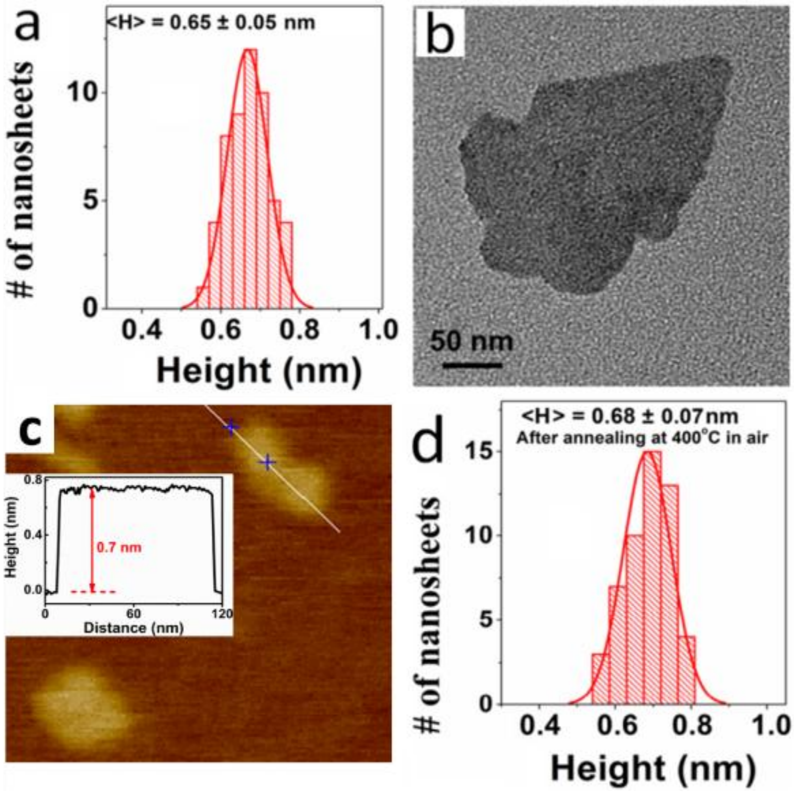

Supplementary Fig. 5 a) Thickness distribution of the fresh Bismuthene sheets measured from AFM images. b) Typical TEM image of Bismuthene sheets after annealing at 400 ^{o}C in air. c) Typical AFM image and the thickness measurement of the annealed Bismuthene sheets. d) Thickness distribution of the annealed Bismuthene sheets measured from the AFM images.

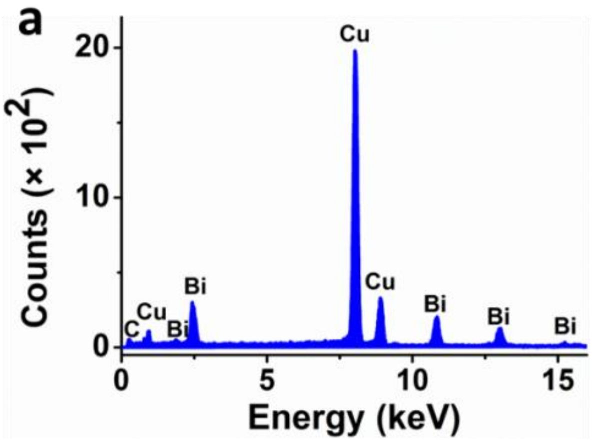

b

Element	Weight %
C	0.32
N	0
O	0
Cu	70.15
Bi	29.53

Supplementary Fig. 6 a) EDS analysis on individual metallic Bismuthene nanosheets. b) The calculated percentages of elements based on (a). The Cu and the small amount of C are from microgrid film of copper-grid.

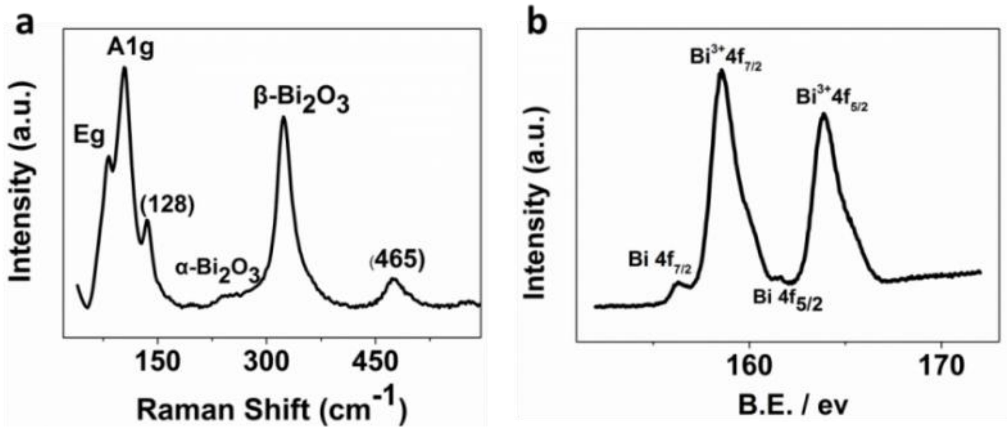

Supplementary Fig. 7 a) Raman spectrum and b) high resolution 4f XPS spectrum of annealed Bismuthene nanosheets.

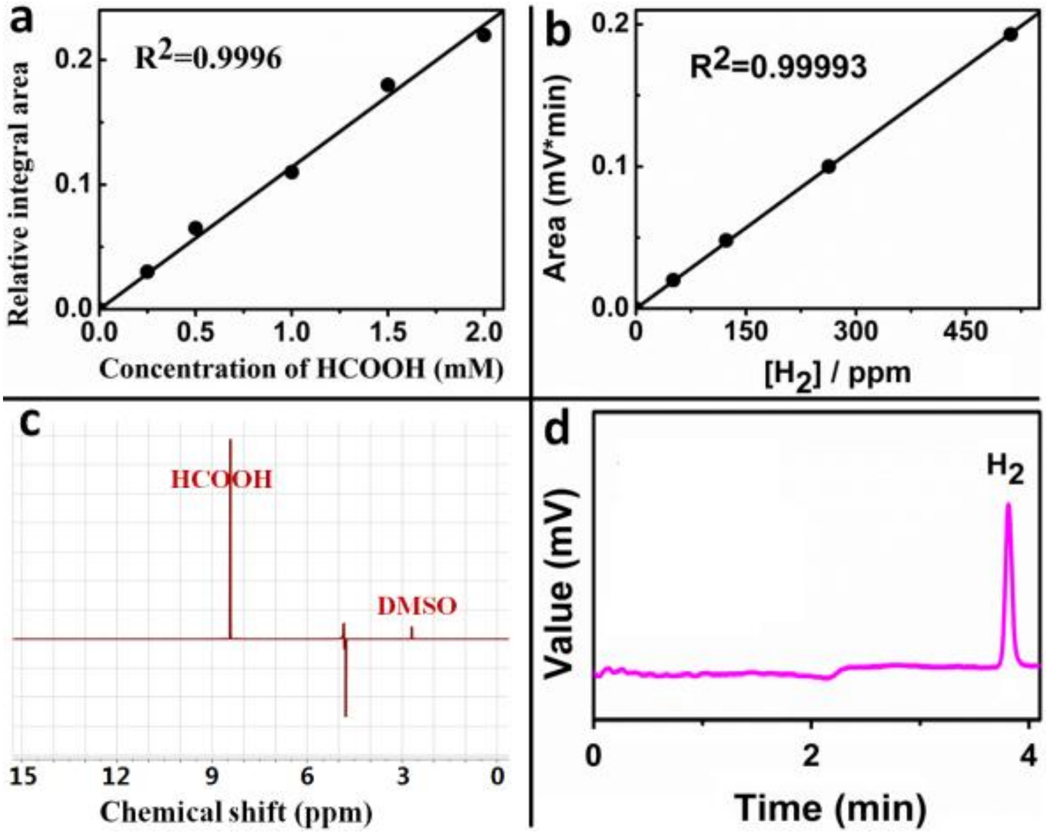

Supplementary Fig. 8 Calibration curves for $HCOO^-$ a), **H_2** b). Based on these calibration curves, the concentrations of reduction products were quantified accurately. c) Representative NMR spectrum of the electrolyte after CO_2 reduction electrolysis at -0.78 V versus RHE for Bismuthene nanosheets. DMSO is used as an internal standard for quantification of $HCOO^-$. d) Representative GC spectrum of the gas phase after CO_2 reduction electrolysis at -0.78 V versus RHE for Bismuthene nanosheets.

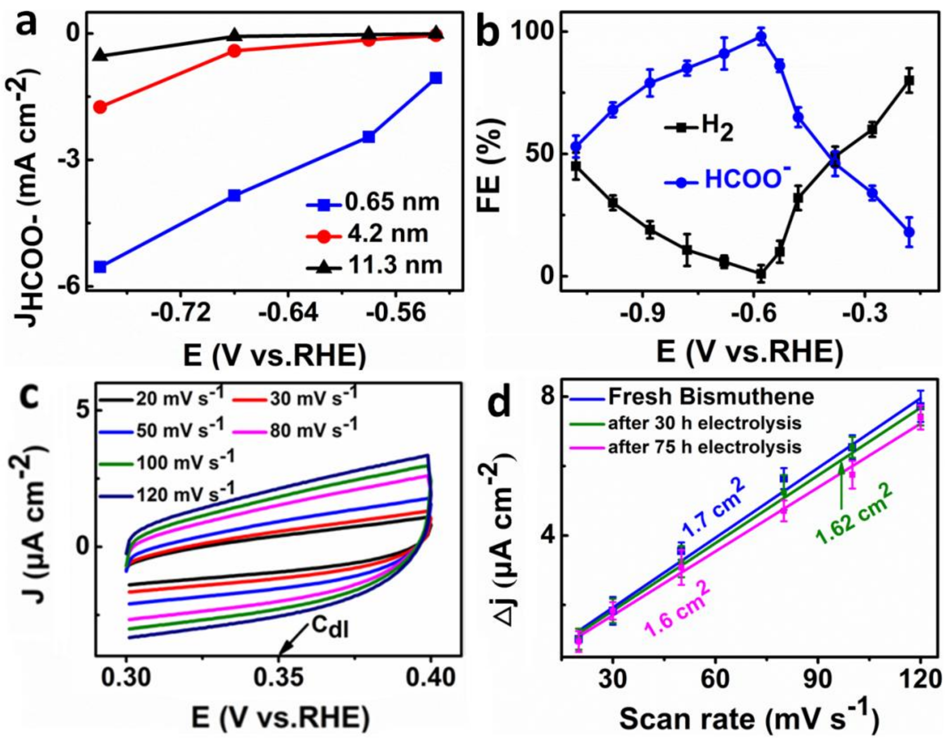

Supplementary Fig. 9 a) Partial current density for $HCOO^-$ (j_{HCOO-}) versus various potentials by BiNSs with different thicknesses. **b**) FEs of $HCOO^-$ and H_2 at various applied potentials on Bismuthene nanosheets. **c**) Cyclic voltammograms of Bismuthene nanosheets within the potential range from -0.4 to -0.3 V (*vs*.RHE) in a N_2-bubbled 0.5 M $KHCO_3$ electrolyte. **d**) ECSA variation of Bismuthene nanosheets after long-term CO2RR operation at -0.58 V. Onset overpotential=E^o-E_{onset} =-0.09 V *vs*.RHE - E_{onset} (V *vs*.RHE). All the error bars in (**b**,**d**) represent the standard error of the mean.

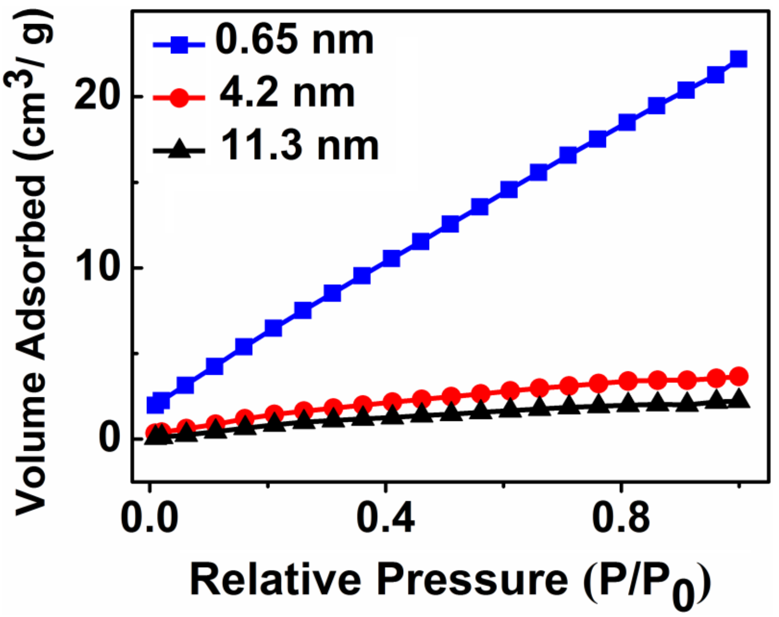

Supplementary Fig. 10 CO_2 adsorption isotherms of BiNSs with different thickness.

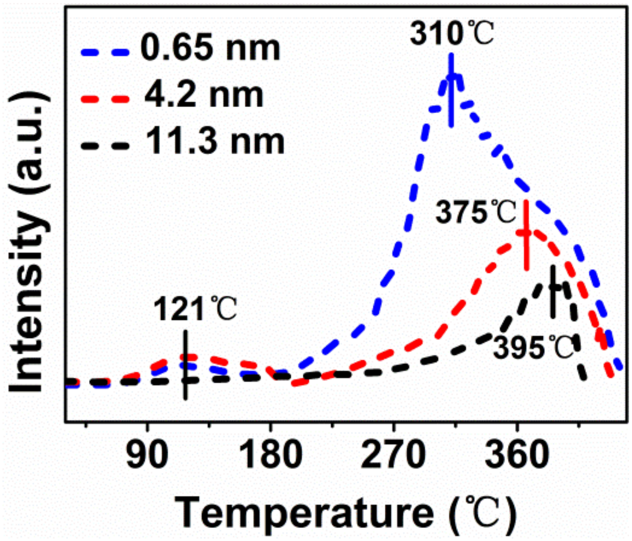

Supplementary Fig. 11 CO_2-TPD spectra on BiNSs with different thicknesses.

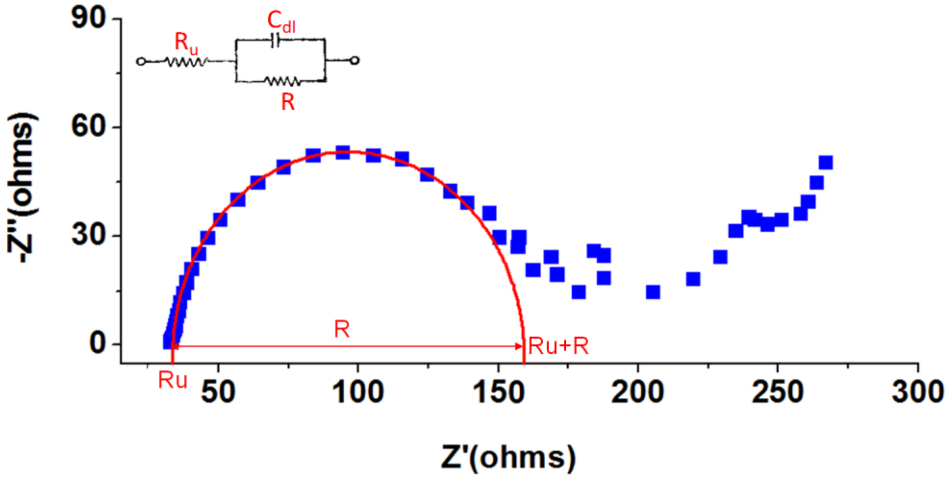

Supplementary Fig. 12 Nyquist plot analysis to obtain the value of resistance (R) of electron transfer on monolayer Bismuthene (0.65 nm) during the CO2RR process.

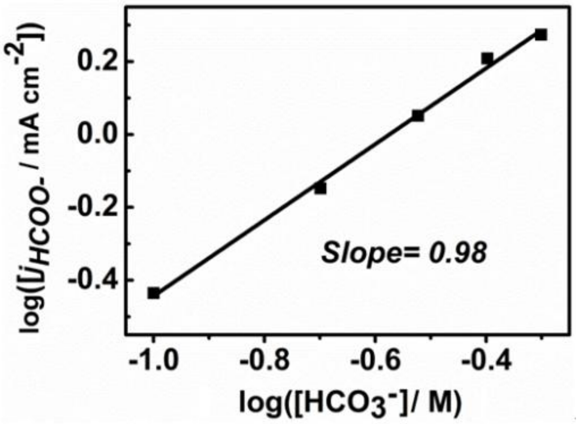

Supplementary Fig. 13 Partial $HCOO^-$ current density of Bismuthene nanosheets *vs* potassium bicarbonate concentration at constant potential of -0.58 V.

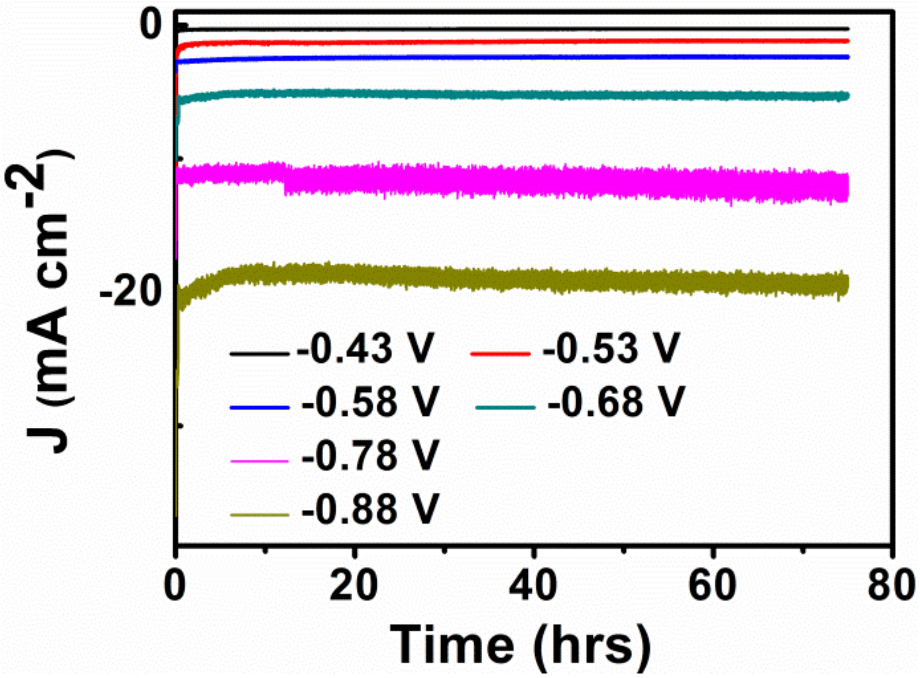

Supplementary Fig. 14 Total CO2RR current density *vs* time of Bismuthene nanosheets catalyst during long-term operation at different applied potentials.

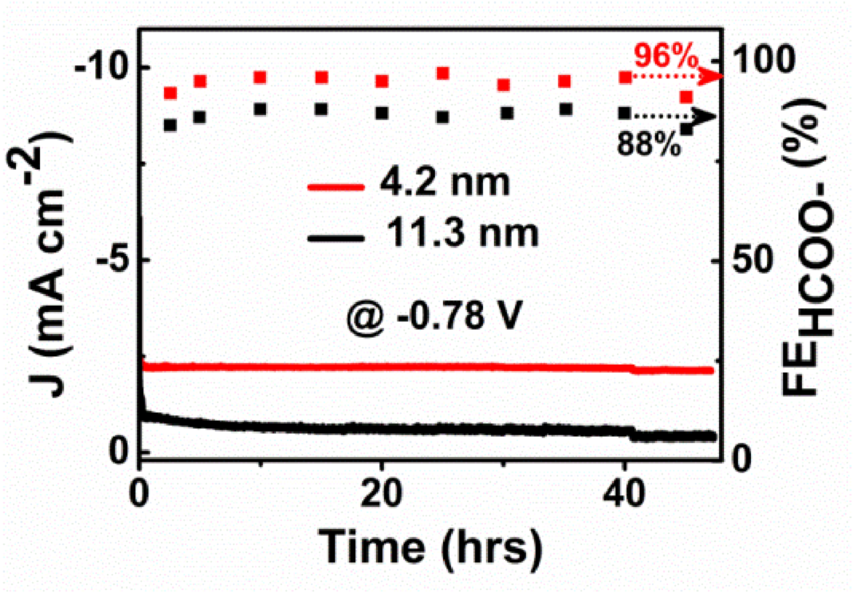

Supplementary Fig. 15 Long-term stability of BiNSs of 4.2 nm and 11.3 nm at a potential of -0.78 V and the corresponding FEs for $HCOO^-$.

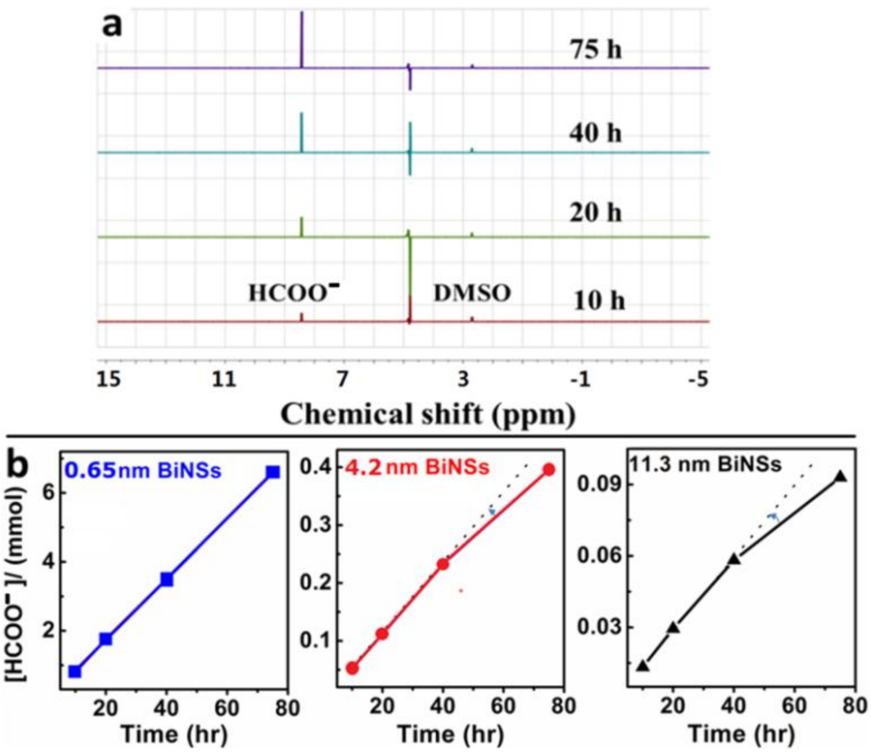

Supplementary Fig. 16 NMR spectra and formate yield. a**)** Representative NMR spectra of the electrolyte after CO2RR at -0.58 V *vs.* RHE for Bismuthene nanosheets. DMSO is used as an internal standard for quantification of $HCOO^-$. b**)** Formate yield in the solution after different CO2RR time for the BiNSs with average thickness of 0.65 nm (left), 4.2 nm (middle) and 11.3 nm (right) at -0.58 V. Independently prepared electrodes were evaluated under identical conditions in b. All data are based on calibration curves.

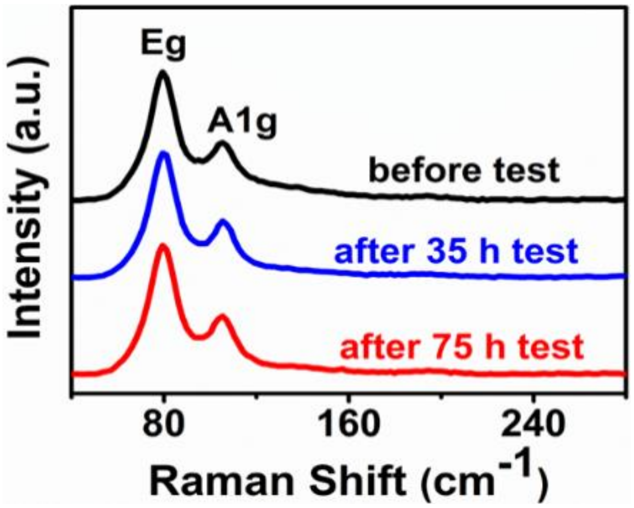

Supplementary Fig. 17 Comparison of Raman spectra for the BiNSs before and after the 35 h and 75 h CO_2 reduction test. The samples for the Raman characterizations were tested as follows: The nanosheets ink was dropped on glassy carbon working electrode, after CO2RR of 35 h or 75 h, the working electrode was directly used for the Raman test to avoid oxidation.

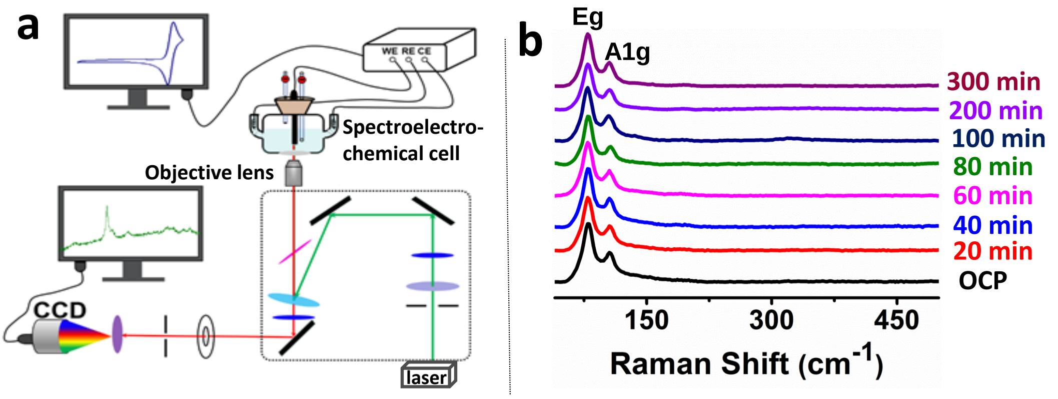

Supplementary Fig. 18 (a) Scheme of *in-situ* SERS set-up and the spectroelectrochemical cell. (b) Real-time *in-situ* Raman spectroscopic of Bismuthene in a CO_2-saturated 0.5 M $KHCO_3$ solution at -0.58 V vs. RHE.

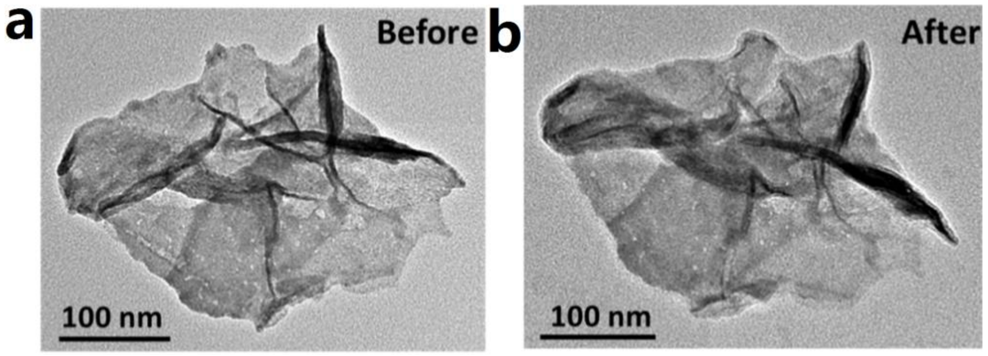

Supplementary Fig. 19 TEM image of the same piece of Bismuthene nanosheet before a) and after b) working 75 h at -0.58 V in CO_2-sautrated $KHCO_3$ solution.

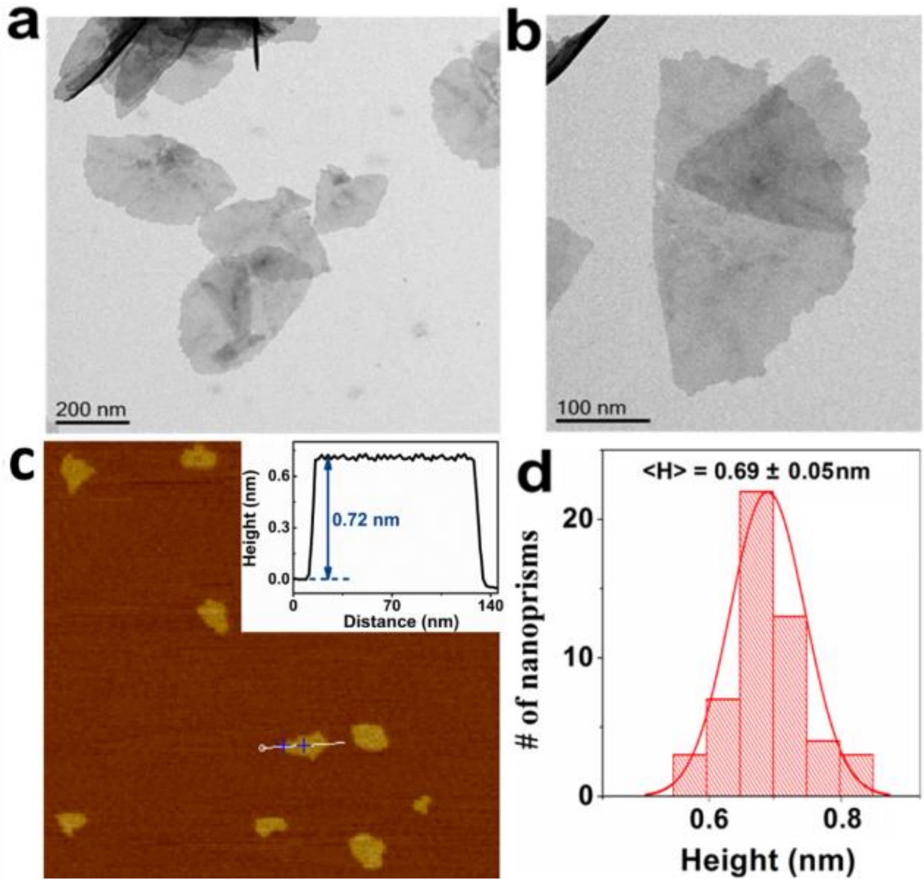

Supplementary Fig. 20 Characterizations for the Bismuthene nanosheets after working 75 h at -0.58 V. a) and b) Typical TEM image of BiNSs. c) Typical AFM image. And d) the corresponding height profiles with average thickness of 0.69 nm after 75 h test. The samples for the above characterizations were produced as follows for both TEM and AFM: the working electrodes after 75 h of electrolysis were sonicated in ethanol for about 30 min and then the samples were collected by centrifuging the mixture, washed with a 2:1 mixture of DI water and absolute ethanol many times, and then dried in vacuum.

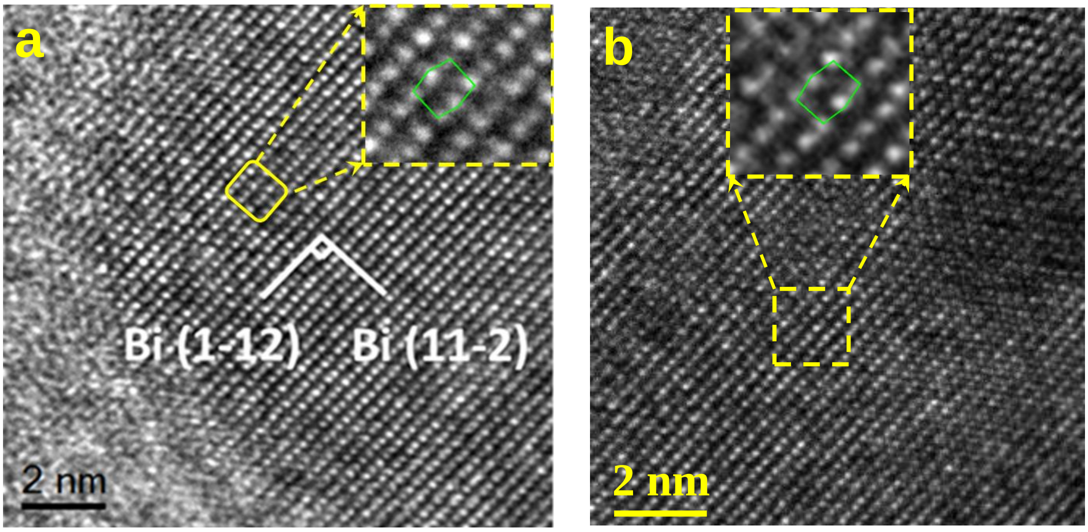

Supplementary Fig. 21 HRTEM-based structural analysis of Bismuthene nanosheets before (a) and after (b) long-term (75 hrs) high current density operation.

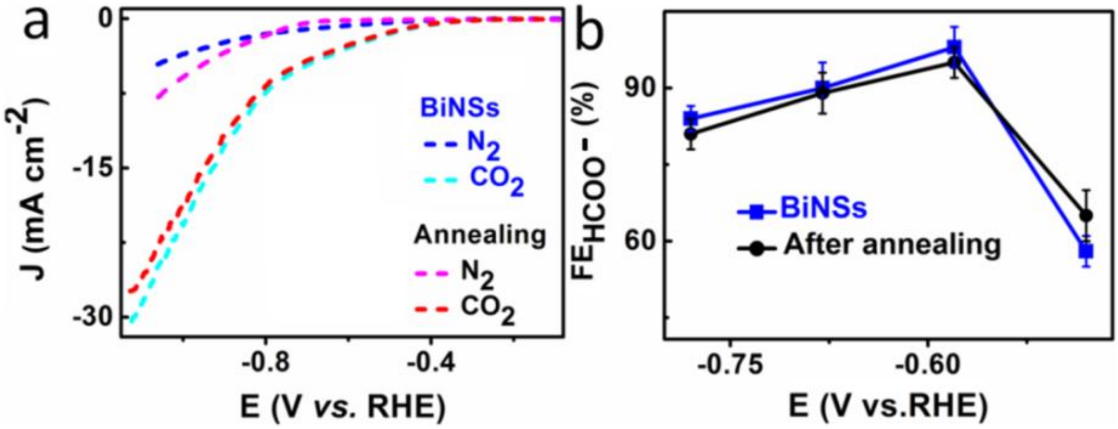

Supplementary Fig. 22 CO2RR performance ((a) LSV and (b) FEs of $HCOO^{-}$) of Bismuthene before and after annealing at 400 °C in air. It shows no CO2RR performance decay after annealing. All the error bars represent the standard error of the mean.

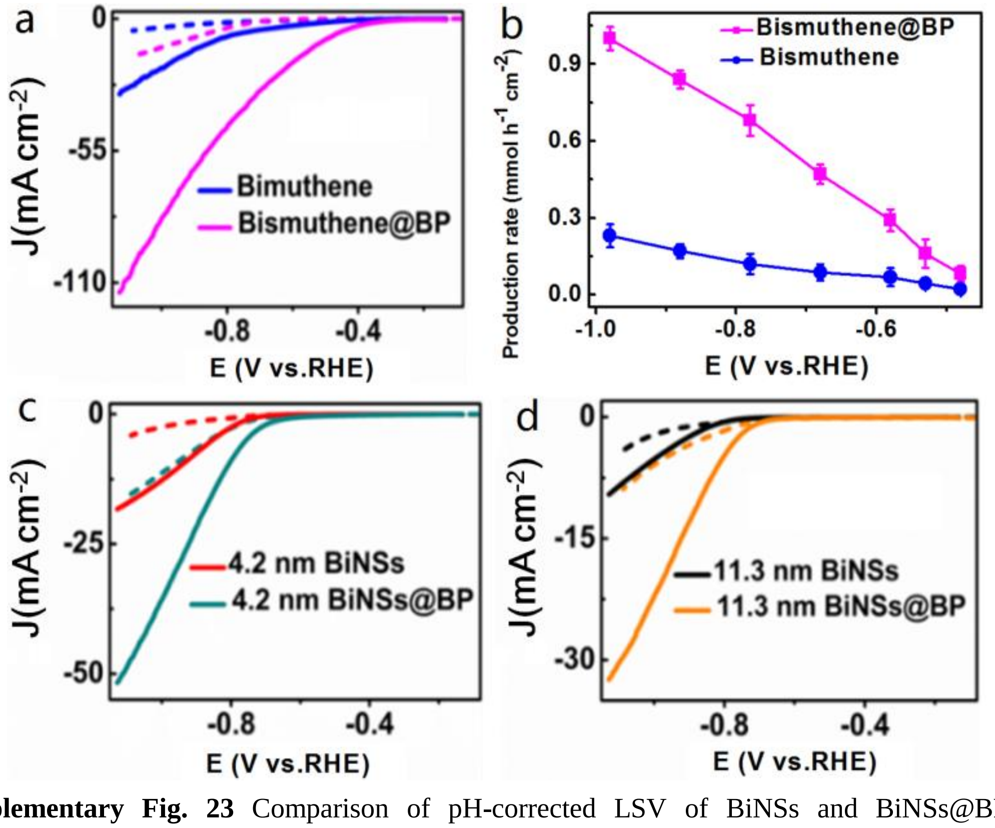

Supplementary Fig. 23 Comparison of pH-corrected LSV of BiNSs and BiNSs@BP in N_2-saturated and CO_2-saturated 0.5 M $KHCO_3$ solution with the same catalysts loading of 0.39 mg/cm^2 on glassy carbon electrode after inserting BP: a) 0.65 nm Bismuthene. c) 4.2 nm BiNSs. d) 11.3 nm BiNSs. As is shown that the current density increased with no change of response to CO_2 after inserting BP. (b) Formate production rates at different potentials on Bismuthene and Bismuthene@BP. All the error bars represent the standard error of the mean.

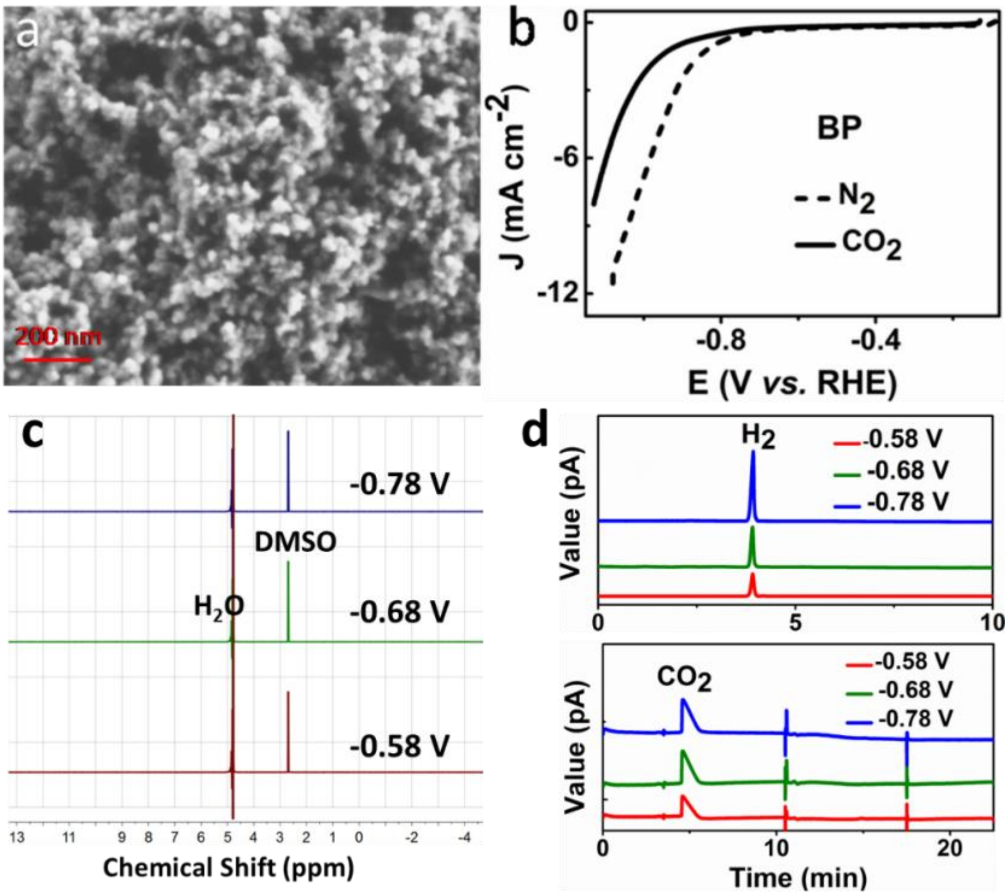

Supplementary Fig. 24 The response of BP to CO2RR. a) Typical SEM image of carbon black BP. b) pH-corrected linear sweep voltammetric curves (LSV) of BP in N_2-saturated and CO_2-saturated 0.5 M $KHCO_3$ solution. c) Representative ^{1}H-NMR spectrum to show no formation of liquid product from CO2RR on BP at different potentials in CO_2-saturated $KHCO_3$. d) GC results (FID detector-based (down) and TCD-based (top)) to show no gas product from CO2RR on BP.

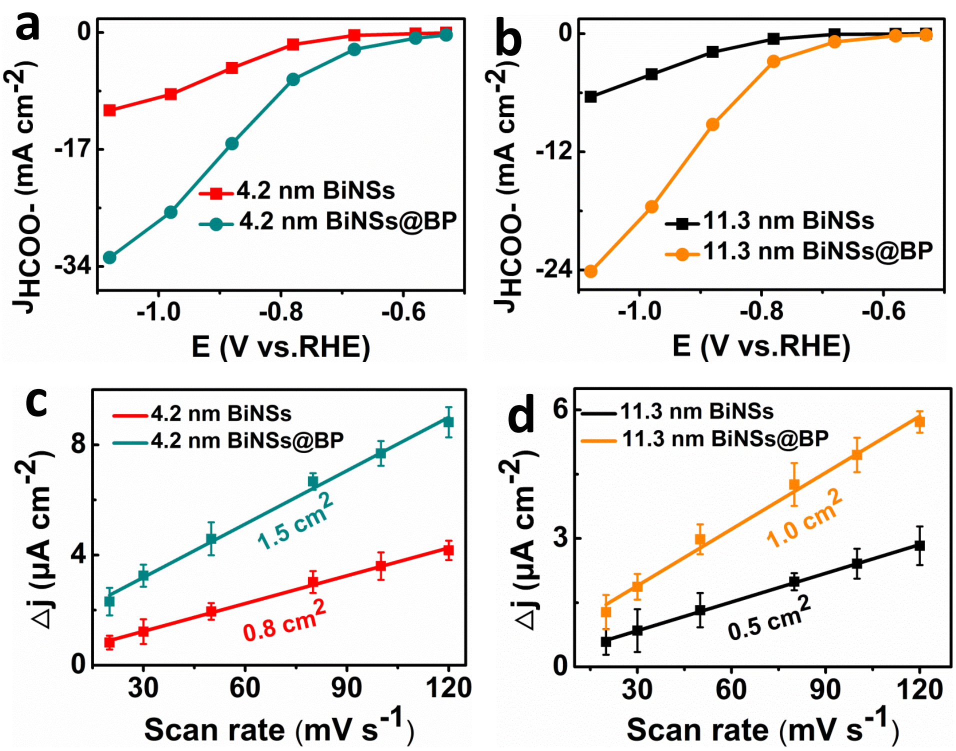

Supplementary Fig. 25 a, b**)** Partial current density for $HCOO^-$ (j_{HCOO^-}) versus potential on thicker BiNSs and BiNSs@BP (with BP 3 wt% optimally). c, d) ECSA measurement for both pure BiNSs and BiNSs@BP. These experimental results further indicate the formation of incompact catalyst layer. All the error bars in (c,d) represent the standard error of the mean.

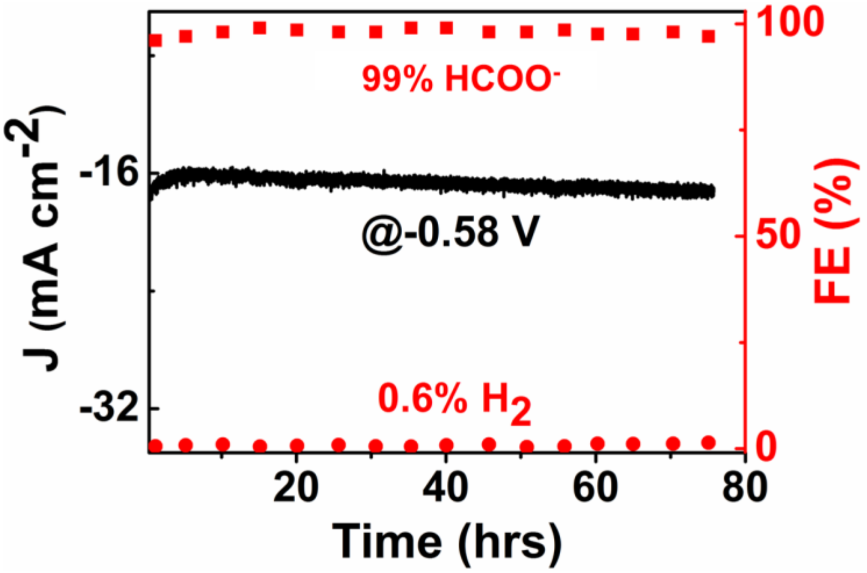

Supplementary Fig. 26 Long-term stability of Bismuthene@BP at a potential of -0.58 V and the corresponding FEs for $HCOO^-$ and H_2.

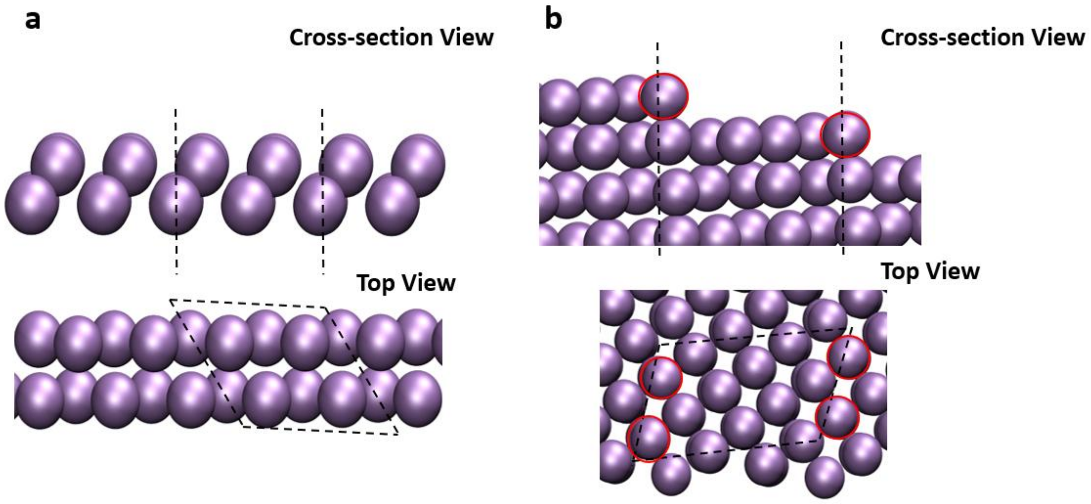

Supplementary Fig. 27 Optimized structures of the Bi(111) one-atom-thick monolayer (a) and thick Bi(011) nanosheets (b) slab model. Top and cross-section views of the structures are provided in the bottom and upper panels, respectively. Bi atoms are represented with violet spheres. Undercoordinated Bi atoms at the step edges of the Bi(011) surface are highlighted in red. Only the top four layers of the Bi(011) slab are shown. Dashed black lines define the unit cell lattice vectors.

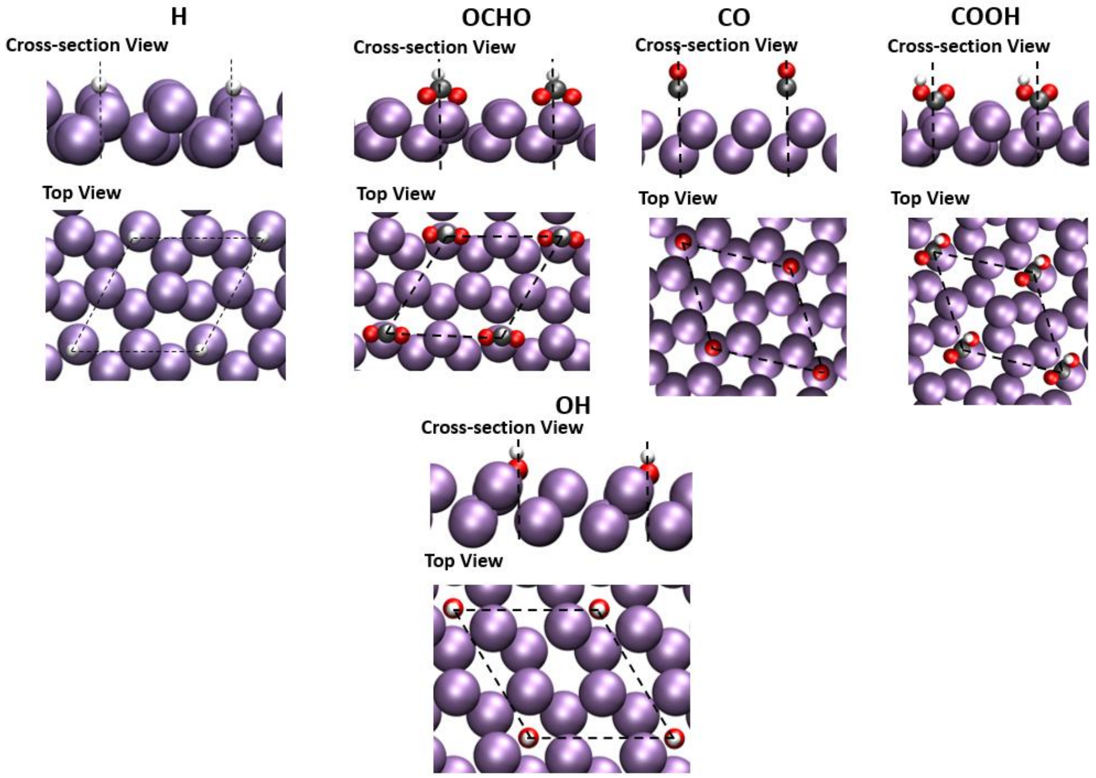

Supplementary Fig. 28 Optimized geometries for the CO2RR and HER intermediates on the Bi(111) single-atom-thick monolayer. Bi, C, O, and H atoms are represented with violet, grey, red, and white spheres, respectively. Top and cross-section views are reported in the bottom and upper panel, respectively. Dashed black lines delineate the unit cell lattice vectors.

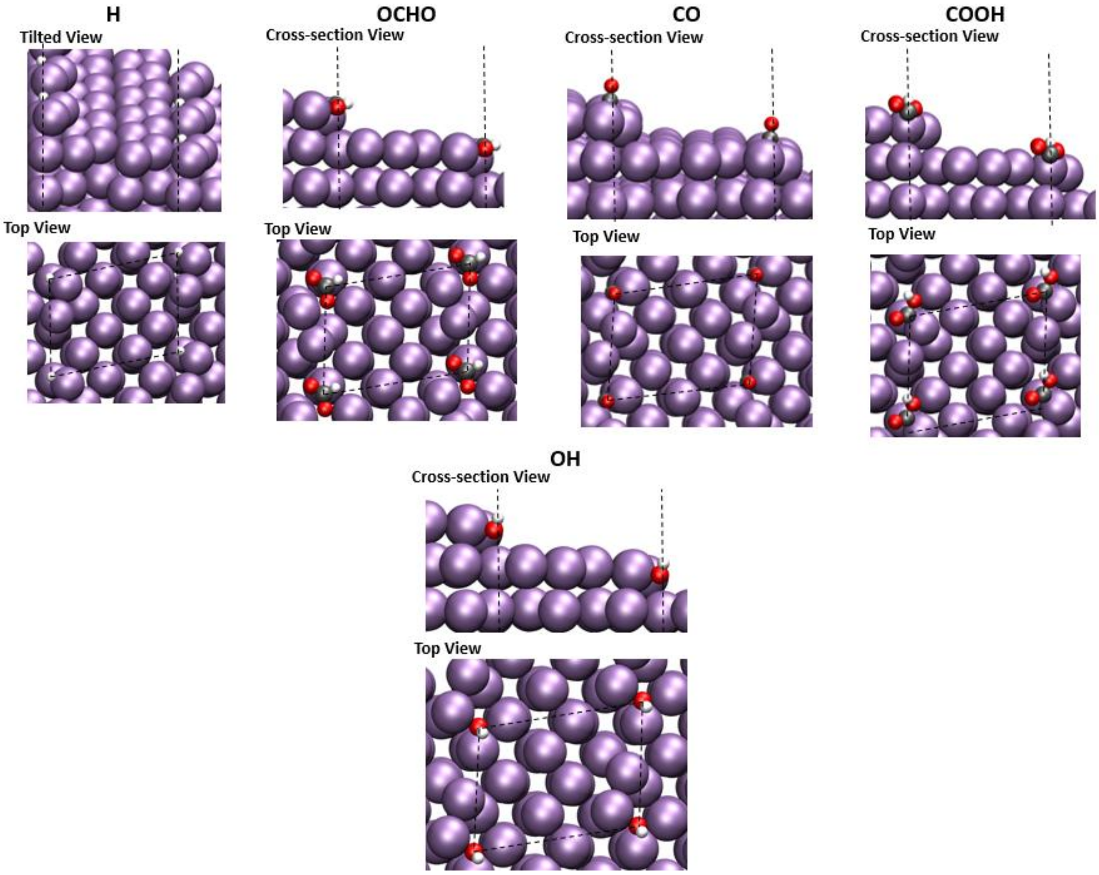

Supplementary Fig. 29 Optimized, most stable adsorption structures for the CO2RR and HER intermediates on the step edge sites of Bi(011) thick nanosheets model. Bi, C, O, and H atoms are represented with violet, grey, red, and white spheres, respectively. Top and cross-section views are provided in the bottom and upper panel, respectively. For H only, a tilted view is given. Dashed black lines delineate the unit cell lattice vectors.

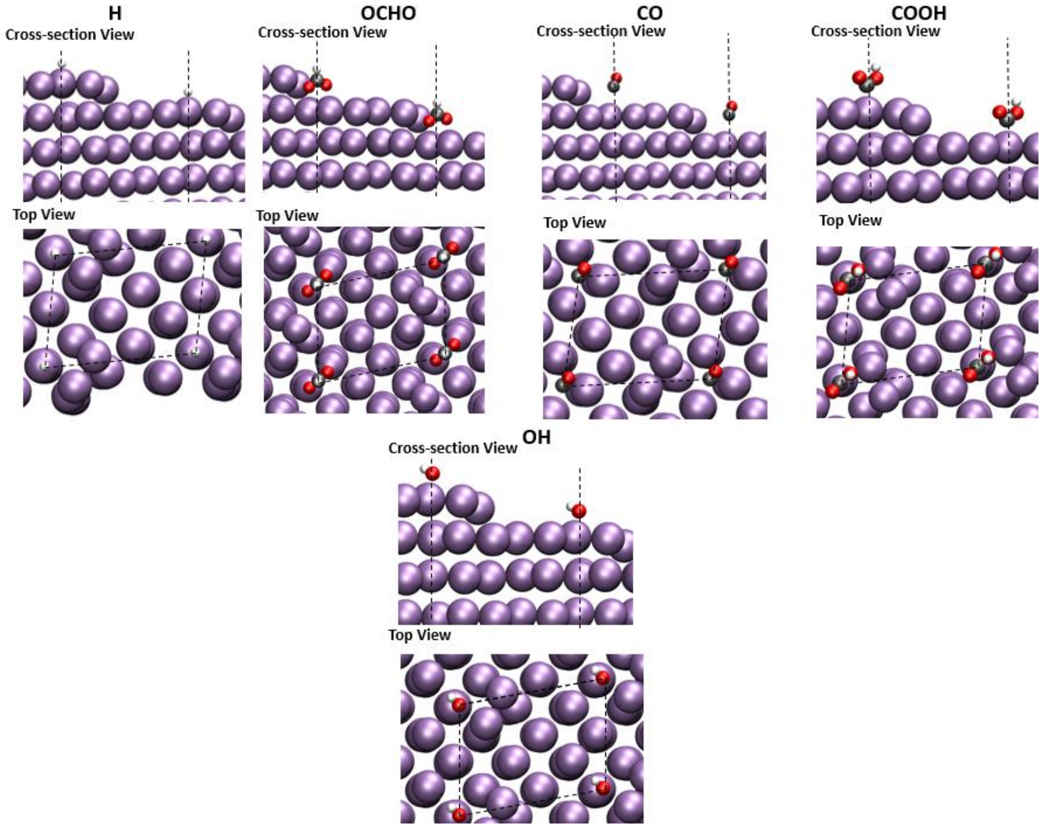

Supplementary Fig. 30 Optimized structures of the CO2RR and HER intermediates on the terrace sites of the Bi(011) slab model for the thick nanosheets. Bi, C, O, and H atoms are represented with violet, grey, red, and white spheres, respectively. Top and cross-section views are provided in the bottom and upper panel, respectively. Dashed black lines delineate the unit cell lattice vectors.

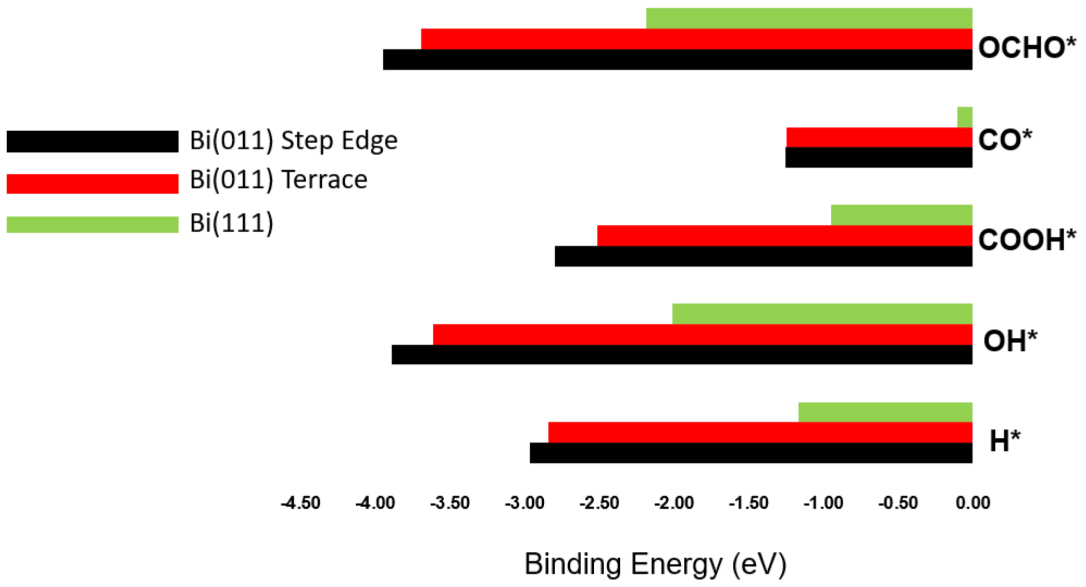

Supplementary Fig. 31 Binding energy of OCHO*, CO*, COOH*, OH*, and H* on the Bi(111) monolayer (green bar), on the step edge sites of the Bi(011) surface (black bar), and on the terrace sites of the Bi(011) surface (red bar). For the Bi(011) model, adsorption is always preferred on the step edges. Therefore, all results reported elsewhere for this model reflect adsorption on step edges, not the terraces. The more negative binding energy reflects stronger interaction. Zero corresponds to the solid model at infinite separation from the gas phase adsorbate species.

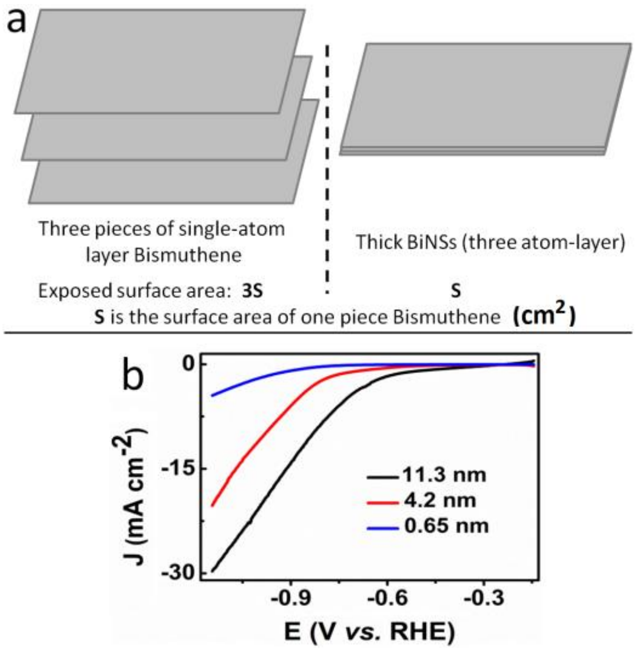

Supplementary Fig. 32 Comparison of the intrinsic HER activity of different BiNSs. a) Scheme for the calculation of exposed surface areas of same amount of BiNSs in different thickness. b) Comparison of pH-corrected LSV of BiNSs in Ar-saturated 0.5 M KH_2PO_4/K_2HPO_4 (pH 7.0) solutions with the same catalysts loading of 0.39 mg/cm^2 on glassy carbon electrode. The current density was calculated based on the exposed real surface area estimated based on the scheme shown in (a).

Supplementary Table 1. The peak potentials and FE_{HCOO^-} of different catalysts for CO2RR.

Catalysts	Electrolyte	peak potential	FE_{HCOO^-}	Reference
**Bismuthene**	**0.5 M $KHCO_3$**	**-0.58 V *vs*.RHE**	**98%**	**This work**
**Bismuthene@BP**	**0.5 M $KHCO_3$**	**-0.58 V *vs*.RHE**	**99%**	**This work**
BiNS	0.5M $NaHCO_3$	-1.5 V *vs*.SCE	95%	Ref. 1
Sn dendrite	0.1 M $KHCO_3$	-1.36 V *vs*.RHE	71.6%	Ref. 2
Bi Dendrite	0.5 M $KHCO_3$	-0.74 V *vs*.RHE	89%	Ref. 3
nano-Bi/Cu	0.1 M $KHCO_3$	-1.5 V *vs*. Ag/AgCl	91.3%	Ref. 4
HSA-Bi	0.5 M $KHCO_3$	-1.5 V *vs*.SCE	92%	Ref. 5
Bi nanodendrite	0.5 M $NaHCO_3$	-1.8 V *vs*.SCE	96.4%	Ref. 6
nano-SnO_2/graphene	0.1 M $NaHCO_3$	-1.8 V *vs*.SCE	93.6%	Ref. 7
Roughened Pb	0.1 M $KHCO_3$	-0.96 V *vs*.RHE	88%	Ref. 8
Partially oxidized Co	0.1 M $NaSO_4$	-0.85 V *vs*.SCE	90%	Ref. 9
Co_3O_4 Layers	0.1 M $KHCO_3$	-0.88 V *vs*.SCE	64.3%	Ref. 10
Mesoporous SnOx	0.1 M $KHCO_3$	-1.15 V *vs*.RHE	75%	Ref. 11
nano-Bi	0.5 M $KHCO_3$	-1.6 V *vs*.SCE	98.4%	Ref. 12
Oxide-derived Bi	0.5 M $KHCO_3$	-0.82 V *vs*.RHE	82%	Ref. 13
PEI-NCNT/GC	0.1 M $KHCO_3$	-1.8 V *vs*.SCE	87%	Ref. 14
Sulfur-Doped Copper	0.1 M $KHCO_3$	-0.8 V *vs*.RHE	75%	Ref. 15
CuO-derived Cu	0.5 M $KHCO_3$	-1.4 V *vs*. Ag/AgCl	61%	Ref. 16
Bi_{45}/GDE	0.5 M $KHCO_3$	-1.45 V *vs*.SCE	90%	Ref. 17
Bi nanosheets	0.1M $KHCO_3$	-1.1 V *vs*.RHE	86%	Ref. 18
Shape-controlled Bi	0.1 M $KHCO_3$	-0.6 V *vs*.RHE	99%	Ref. 19
reduced mpBi nanosheets	0.5 M $NaHCO_3$	-0.9 V *vs*.RHE	99%	Ref. 20
Cu foam@BiNW	0.5 M $NaHCO_3$	-0.69 V *vs*.RHE	95%	Ref. 21
Bi_2O_3-NGQDs	0.5 M $KHCO_3$	-0.9 V *vs*.RHE	100%	Ref. 22
Bi_2O_3NSs@MCCM	0.1 M $KHCO_3$	-1.256 V *vs*.RHE	93.8%	Ref. 23
POD-Bi	0.5 M $KHCO_3$	-1.16 V *vs*.RHE	95%	Ref. 24

E^o ($CO_2/HCOO^-$) = -0.09 V (vs. RHE in neutral solutions, *Nano Energy* **31**, 270-277 (2017)).

Supplementary Table 2. Zero-point energies (ZPE), entropic (T*S), and solvation corrections used in calculating the free energies (G) of surface intermediates on both model surfaces. Entries for (111) refer to results on the single-atom-thick bismuthene model, and entries for (011) refer to results on the thick NS Bi(011) model.

	ZPE (eV)		T*S (eV)		Solvation (eV)	
	(111)	(011)	(111)	(011)	(111)	(011)
CO*	0.15	0.15	0.32	0.33	0.03	0.04
OH*	0.33	0.33	0.11	0.11	-0.05	-0.10
COOH*	0.59	0.59	0.25	0.25	-0.19	-0.17
OCHO*	0.59	0.59	0.28	0.30	-0.09	-0.06
H*	0.15	0.15	0.02	0.02	0.00	0.03

Supplementary References

1. Han, N., Wang, Y., Yang, H., Deng, J., Wu, J., Li, Y. & Li, Y. Ultrathin bismuth nanosheets from in situ topotactic transformation for selective electrocatalytic CO_2 reduction to formate. *Nat. Commun.* **9**, 1320 (2018).

2. Won, D. H., Choi, C. H., Chung, J., Chung, M. W., Kim, E.-H. & Woo, S. I. Rational Design of a Hierarchical Tin Dendrite Electrode for Efficient Electrochemical Reduction of CO_2. *ChemSusChem* **8**, 3092–3098 (2015).

3. Koh, J. H., Won, D. H., Eom, T., Kim, N.-K., Jung, K. D., Kim, H., Hwang, Y. J. & Min, B. K. Facile CO_2 Electro-Reduction to Formate via Oxygen Bidentate Intermediate Stabilized by High-Index Planes of Bi Dendrite Catalyst. *ACS Catal.* **7**, 5071–5077 (2017).

4. Lv, W., Zhou, J., Bei, J., Zhang, R., Wang, L., Xu, Q. & Wang, W. Electrodeposition of nano-sized bismuth on copper foil as electrocatalyst for reduction of CO_2 to formate. *Applied Surf. Sci.* **393**, 191–196 (2017).

5. Zhang, H., Ma, Y., Quan, F., Huang, J., Jia, F. & Zhang, L. Selective electro-reduction of CO_2 to formate on nanostructured Bi from reduction of BiOCl nanosheets. *Electrochem. Commun.* **46**, 63–66 (2014).

6. Zhong, H., Qiu, Y., Zhang, T., Li, X., Zhang, H. & Chen, X. Bismuth nanodendrites as a high performance electrocatalyst for selective conversion of CO_2 to formate. *J. Mater. Chem. A* **4**, 13746–13753 (2016).

7. Zhang, S., Kang, P. & Meyer, T. J. Nanostructured Tin Catalysts for Selective Electrochemical Reduction of Carbon Dioxide to Formate. *J. Am. Chem. Soc.* **136**, 1734–1737 (2014).

8. He, Z., Shen, J., Ni, Z., Tang, J., Song, S., Chen, J. & Zhao, L. Electrochemically created roughened lead plate for electrochemical reduction of aqueous CO_2. *Catal. Commun.* **72**, 38–42 (2015).

9. Gao, S., Lin, Y., Jiao, X., Sun, Y., Luo, Q., Zhang, W., Li, D., Yang, J. & Xie, Y. Partially oxidized atomic cobalt

layers for carbon dioxide electroreduction to liquid fuel. *Nature* **529**, 68–71 (2016).

10. Gao, S., Jiao, X., Sun, Z., Zhang, W., Sun, Y., Wang, C., Hu, Q., Zu, X., Yang, F., Yang, S., Liang, L., Wu, J. & Xie, Y. Ultrathin Co_3O_4 Layers Realizing Optimized CO_2 Electroreduction to Formate. *Angew. Chem.* **128**, 708–712 (2016).

11. Daiyan, R., Lu, X., Saputera, W. H., Ng, Y. H. & Amal, R. Highly Selective Reduction of CO_2 to Formate at Low Overpotentials Achieved by a Mesoporous Tin Oxide Electrocatalyst. *ACS Sustainable Chem. Eng.* **6**, 1670–1679 (2018).

12. Qiu, Y., Du, J., Dong, W., Dai, C. & Tao, C. Selective conversion of CO_2 to formate on a size tunable nano-Bi electrocatalyst. *J. of CO_2 Utilization* **20**, 328–335 (2017).

13. Bertin, E., Garbarino, S., Roy, C., Kazemi, S. & Guay, D. Selective electroreduction of CO_2 to formate on Bi and oxide-derived Bi films. *J. of CO_2 Utilization* **19**, 276–283 (2017).

14. Zhang, S., Kang, P., Ubnoske, S., Brennaman, M. K., Song, N., House, R. L., Glass, J. T. & Meyer, T. J. Polyethylenimine-Enhanced Electrocatalytic Reduction of CO_2 to Formate at Nitrogen-Doped Carbon Nanomaterials. *J. Am. Chem. Soc.* **136**, 7845–7848 (2014).

15. Huang, Y., Deng, Y., Handoko, A. D., Goh, G. K. L. & Yeo, B. S. Rational Design of Sulfur-Doped Copper Catalysts for the Selective Electroreduction of Carbon Dioxide to Formate. *ChemSusChem* **11**, 320–326 (2018).

16. Gupta, K., Bersani, M. & Darr, J. A. Highly efficient electro-reduction of CO_2 to formic acid by nano-copper. *J. Mater. Chem. A* **4**, 13786–13794 (2016).

17. Zhang, X., Lei, T., Liu, Y. & Qiao, J. Enhancing CO_2 electrolysis to formate on facilely synthesized Bi catalysts at low overpotential. *Appl. Cat. B: Environ.* **218**, 46–50 (2017).

18. Zhang, W., Hu, Y., Ma, L., Zhu, G., Zhao, P., Xue, X., Chen, R., Yang, S., Ma, J., Liu, J. & Jin, Z. Liquid-phase exfoliated ultrathin Bi nanosheets: Uncovering the origins of enhanced electrocatalytic CO_2 reduction on two-dimensional metal nanostructure. *Nano Energy* **53**, 808–816 (2018).

19. Kim, S., Dong, W. J., Gim, S., Sohn, W., Park, J. Y., Yoo, C. J., Jang, H. W. & Lee, J.-L. Shape-controlled bismuth nanoflakes as highly selective catalysts for electrochemical carbon dioxide reduction to formate. *Nano Energy* **39**, 44–52 (2017).

20. Yang, H. et al. Selective CO_2 Reduction on 2D Mesoporous Bi Nanosheets. *Adv. Energy Mater.* **8**, 1801536 (2018).

21. Zhang, X., Sun, X., Guo, S.-X., Bond, A. M. & Zhang, J. Formation of lattice-dislocated bismuth nanowires on

copper foam for enhanced electrocatalytic CO_2 reduction at low overpotential. *Energy Environ. Sci.* **12**, 1334-1340 (2019).

22. Chen, Z., Mou, K., Wang, X. & Liu, L. Nitrogen-Doped Graphene Quantum Dots Enhance the Activity of Bi_2O_3 Nanosheets for Electrochemical Reduction of CO_2 in a Wide Negative Potential Region. *Angew. Chem. Int. Ed.* **57**, 12790–12794 (2018).
23. Liu, S., Lu, X. F., Xiao, J., Wang, X. & Lou, X. W. Bi_2O_3 Nanosheets Grown on Multi-Channel Carbon Matrix to Catalyze Efficient CO_2 Electroreduction to HCOOH. *Angew. Chem. Int. Ed*. **58**, 13828-13833 (2019).
24. He, S. et al. The p-Orbital Delocalization of Main-Group Metals to Boost CO_2 Electroreduction. *Angew. Chem. Int. Ed*. **130**, 16346-16351 (2018).
